# The Four-Feature Prognostic Models for Cancer-Specific and Overall Survival after Surgery for Localized Clear Cell Renal Cancer: Is There a Place for Inflammatory Markers?

**DOI:** 10.3390/biomedicines10051202

**Published:** 2022-05-23

**Authors:** Łukasz Zapała, Aleksander Ślusarczyk, Rafał Wolański, Paweł Kurzyna, Karolina Garbas, Piotr Zapała, Piotr Radziszewski

**Affiliations:** Clinic of General, Oncological and Functional Urology, Medical University of Warsaw, Lindleya 4, 02-005 Warsaw, Poland; rafalwolanski7@gmail.com (R.W.); paw.kurzyna@gmail.com (P.K.); trelkowa98@gmail.com (K.G.); zapala.piotrek@gmail.com (P.Z.); pradziszewski@wum.edu.pl (P.R.)

**Keywords:** renal cell carcinoma, risk models, survival analysis, charlson comorbidity index, systemic inflammatory markers

## Abstract

We aimed at a determination of the relevance of comorbidities and selected inflammatory markers to the survival of patients with primary non-metastatic localized clear cell renal cancer (RCC). We retrospectively analyzed data from a single tertiary center on 294 patients who underwent a partial or radical nephrectomy in the years 2012–2018. The following parameters were incorporated in the risk score: tumor stage, grade, size, selected hematological markers (SIRI—systemic inflammatory response index; SII—systemic immune-inflammation index) and a comorbidities assessment tool (CCI—Charlson Comorbidity Index). For further analysis we compared our model with existing prognostic tools. In a multivariate analysis, tumor stage (*p* = 0.01), tumor grade (*p* = 0.03), tumor size (*p* = 0.006) and SII (*p* = 0.02) were significant predictors of CSS, while tumor grade (*p* = 0.02), CCI (*p* = 0.02), tumor size (*p* = 0.01) and SIRI (*p* = 0.03) were significant predictors of OS. We demonstrated that our model was characterized by higher accuracy in terms of OS prediction compared to the Leibovich and GRANT models and outperformed the GRANT model in terms of CSS prediction, while non-inferiority to the VENUSS model was revealed. Four different features were included in the predictive models for CSS (grade, size, stage and SII) and OS (grade, size, CCI and SIRI) and were characterized by adequate or even superior accuracy when compared with existing prognostic tools.

## 1. Introduction

The routine management of localized renal cell cancer (RCC) is radical or partial nephrectomy [1]. However, it should be emphasized that approximately 20–40% of cases become metastatic during the course of the disease, even given successful initial treatment [2]. Therefore, determining the key factors that affect postsurgical prognosis would allow early risk-stratification.

Oncological outcomes are routinely estimated based on the TNM classification and pathological features of a tumor [3]. According to the American Urological Association, the establishment of a prognosis should rely on TNM staging, while localized disease is connected with nearly 90% of cancer-specific survival [4]. On the other hand, there is a strong recommendation from the European Urological Association (EAU) to focus on more sophisticated tools along with a statement that new models should be compared to already existing tools prior to their introduction into the clinic [5,6]. Although it is obviously included in all the models, TNM staging proved to have restricted accuracy if selected as a single prognostic factor [2]. Additional information has been routinely gathered from pathological examination, i.e., grading or presence of tumor necrosis or sarcomatoid features [2].

Among other clinical parameters, a prognosis is often assessed using gender [7] or age [8]. It is thought that male patients may present with worse prognoses, similar to elderly people [7,8]. Interestingly, the observation that comorbidities may have even greater significance is often connected with the fact that urological cancers are diagnosed frequently in the geriatric population [3]. Consequently, despite curative surgery, other causes of mortality in RCC cases may be of crucial importance.

Among several others, three models have been validated in the literature on localized RCC, i.e., VENUSS (VEnous extension, NUclear grade, Size, Stage) [9], GRANT (GRade, Age, Nodes and Tumor) [10] and Leibovich (tumor stage, regional lymph node status, tumor size, nuclear grade and histologic tumor necrosis) [11]. Although their prognostic accuracies have already been documented, they are not commonly implemented in everyday practice. Furthermore, the EAU does not place one particular tool above the others and leaves the choice up to the clinician.

One can observe a recent growing interest in the novel inflammatory markers that can be easily obtained from preoperative complete blood counts and incorporated into clinical models for prognostic purposes [12]. Recently, we compared the accuracy of different inflammatory markers in the prognostic assessment of RCC and proved that these clinical parameters may enrich existing models [13]. However, the common clinicopathological features remained the pillars of risk stratification, as described by other authors [14].

The aim of the present study was to determine the relevance of comorbidities and selected inflammatory markers to the survival of patients with localized RCC treated by partial and radical nephrectomy in the search for a prognostic model. Here, we focused on a cohort of clear cell RCC patients as a predominant subtype, taking into consideration that the majority of clinical trials enroll these particular patients. The identification of the most efficient model seems to be of greatest importance in terms of both recruiting for future clinical trials and identifying the optimal candidates for adjuvant therapy.

## 2. Materials and Methods

### 2.1. Study Population

We collected and retrospectively analyzed the data from a single tertiary center on patients who underwent partial or radical nephrectomy in the years 2012–2018. We identified 645 patients treated with surgery due to renal cell carcinoma (RCC). We excluded patients who underwent nephrectomy for papillary RCC (*n* = 97) or chromophobe RCC (*n* = 31) or metastatic RCC (*n* = 46) and individuals with missing baseline clinical data (*n* = 138) or who were lost to follow-up shortly after surgery (*n* = 39). Finally, 294 patients with primary non-metastatic localized and locally advanced clear cell carcinoma treated with nephrectomy in our center were enrolled for further analyses.

Information about demographic, clinical and pathological features were collected. No previous cancer management was initiated prior to the radical surgery in all cases. The following clinical parameters were obtained: (a) demographic: age, sex; (b) body mass index (BMI), clinical staging based on available imaging, i.e., computed tomography or magnetic resonance of chest, abdomen and pelvis according to the 2017 TNM classification [15], comorbidities (including diabetes, hypertension, heart disease, autoimmune diseases) and Charlson Comorbidity Index (CCI) calculated according to Charlson ME et al. [16], chronic drug uptake (statins and beta-adrenolytics), preoperative laboratory findings of full blood count, surgery type (partial or radical nephrectomy); and (c) pathological: tumor stage and histological diagnosis, including grading and presence of potential necrosis or sarcomatoid components (according to Fuhrman and/or WHO/ISUP, when adequate) of clear cell carcinoma.

In the further analyses, we included two most promising inflammatory markers based on neutrophil, lymphocyte, platelet or monocyte counts, i.e., systemic inflammatory response index—SIRI—and systemic immune-inflammation index—SII. Based on the c-indexes obtained in the preliminary calculations and our previous paper on inflammatory biomarkers [13], we included SIRI and SII in the respective survival models. SIRI was calculated as follows: SIRI = neutrophil × monocyte/lymphocyte; SII was calculated as: SII = neutrophil × platelet/lymphocyte. Prior to the construction of a local risk model for patient survival after radical surgery, we performed analyses aimed at the validation of the available tools designed to be used in localized settings, i.e., VENUSS [9], GRANT [10] and Leibovich [11].

Information on cancer-specific survival (CSS), overall survival (OS) and recurrence-free survival (RFS) was included in the follow-up analysis. The study was conducted under the Ethics Committee vote AKBE/72/2021 of the Medical University of Warsaw. All patients signed informed consent.

### 2.2. Statistical Analysis

Statistical analysis was performed in SAS software (version 9.4., SAS Institute Inc., Cary, NC, USA). Baseline patient characteristics were presented as medians with interquartile ranges for continuous variables and numbers with percentages for categorized variables. Differences in continuous variables were compared using the Mann–Whitney U test, while categorized variables were evaluated with Fisher’s exact test. Categorization of continuous variables (e.g., Charlson Comorbidity Index, SIRI, SII) was performed using the optimal cut-off values based on receiver operating curve (ROC) statistics. Logistic regression was utilized for uni- and multivariate analyses. Univariate analyses provided factors for stepwise selection in the development of the multivariate model. Odds ratios with 95% confidence intervals were derived via logistic regression. Two-sided *p*-values < 0.05 denoted statistical significance.

The VENUSS, GRANT and Leibovich models were externally validated with our patient sample using logistic regression and respective area under the curves with c-index for each risk model. The accuracy of the above risk scores was compared with our newly derived model. The differences in survival according to the risk classification based on the VENUSS, GRANT and Leibovich tools and our risk score were analyzed using the Kaplan–Meier method and evaluated with log-rank tests.

## 3. Results

### 3.1. Basic Characteristics of the Cohort

The majority of the cohort were males (n = 185) (please refer to Table 1). The patients were diagnosed mainly with T1 tumors (83.7%) of Fuhrman 1–2 (86%) grade. As for CCI, we stratified patients into ≤4 (57%) and >4 points. The greater percentage of patients underwent partial nephrectomy (65%). The details of patients’ comorbidities are presented in Table 1 in detail. Additionally, a division into different risk groups according to the Leibovich, GRANT and VENUSS models is also presented in Table 1. For the median follow-up period of 53 months (IQR 42.5–61), patient survival values were determined: CSS, 94.6%; OS, 89%; and RFS, 86%.

### 3.2. Univariate and Multivariate Analyses of Factors Predictive for Cancer-Specific Survival in Patients with Clear Cell Renal Cell Carcinoma (ccRCC)

Associations between CSS and clinicopathological variables or laboratory parameters are shown in Table 2 and Table 3. Univariate analysis revealed associations between CSS and high SII (*p* = 0.0025), high SIRI (*p* = 0.0115), tumor grade (*p* < 0.001), tumor stage (*p* < 0.001), tumor size ≥ 7 cm (*p* < 0.001) and surgery type (*p* = 0.0006) (Table 2). In multivariate analysis, tumor stage (*p* = 0.0128), tumor grade (*p* = 0.0354), tumor size (*p* = 0.0063) and SII (*p* = 0.0262) were significant predictors of CSS (Table 3).

### 3.3. Univariate and Multivariate Analyses of Factors Predictive for Overall Survival in Patients with Clear Cell Renal Cell Carcinoma (ccRCC)

In the univariate analysis, the following associations between OS and clinicopathologic or laboratory factors were revealed: age (*p* = 0.03), CCI > 4 (*p* = 0.045), high SII (*p* = 0.033), high SIRI (*p* = 0.006), tumor grade (*p* = 0.0006), tumor stage (*p* = 0.0005), tumor size ≥ 7 cm (*p* = 0.0002) and type of surgery (*p* = 0.02). We failed to confirm the associations between survival and statins and beta-adrenolytics uptake (Table 4). In the multivariate analysis we found that tumor grade (*p* = 0.0265), CCI (*p* = 0.0293), tumor size (*p* = 0.0156) and SIRI (*p* = 0.0334) were significant predictors of OS (Table 5).

### 3.4. Multivariate Analyses of Factors Predictive for Recurrence-Free-Specific Survival in Patients with Clear Cell Renal Cell Carcinoma (ccRCC)

In the multivariate analysis, tumor grade (*p* = 0.004) and tumor size (*p* = 0.0015) were the only variables associated significantly with RFS (Table 6). No statistical significance was noted for CCI or any hematological marker.

### 3.5. Proposal of Novel Scoring System for the Risk of Cancer-Specific Death (CSD) and Overall Mortality (OM) after Surgical Treatment of Clear Cell Renal Cell Carcinoma

The following parameters were incorporated in the risk score assessment (Table 7):

- Tumor stage T1–T2 vs. T3–T4;
- Low grade (G1–2) vs. high grade (G3–4);
- Tumor size (<7 cm vs. ≥7 cm);
- SIRI > 2.15 vs. ≤ 2.15 or SII > 660 vs. ≤ 660;
- CCI > 4 vs. ≤4.

Patients received one point for each unfavorable feature (T3–T4, high-grade, tumor size ≥ 7 cm and SII > 660), as for cancer-specific death analysis, for high-grade tumor, tumor size ≥ 7 cm, SIRI > 2.15 and CCI for overall mortality. Then, patients were stratified into low- (0 points), intermediate- (1–2 points) or high- (3–4 points) risk groups. Using respective stratification, we found that the cohort was comprised of mainly low- and intermediate-risk individuals (CSD—92%, OM—93.5%) (Table 8).

**Table 7:** Scoring system for the risk of cancer-specific death (CSD) and overall mortality (OM) after surgical treatment of clear cell renal cell carcinoma. n/a—not applicable.

	Scoring System	
Variable	CSD	OM
	Score	Score
Stage		
T1–T2	0	n/a
T3–T4	1	n/a
Grade		
Low-grade	0	0
High-grade	1	1
Tumor size		
<7 cm	0	0
≥7 cm	1	1
SIRI		
≤2.15	n/a	0
>2.15	n/a	1
SII		
≤660	0	n/a
>660	1	n/a
Charlson Comorbidity Index		
≤4	n/a	0
>4	n/a	1
Risk group		
Low	0	0
Intermediate	1–2	1–2
High	3–4	3–4

### 3.6. Cancer-Specific Survival According to Risk Stratification in Local and External Models

**Figure 1:**
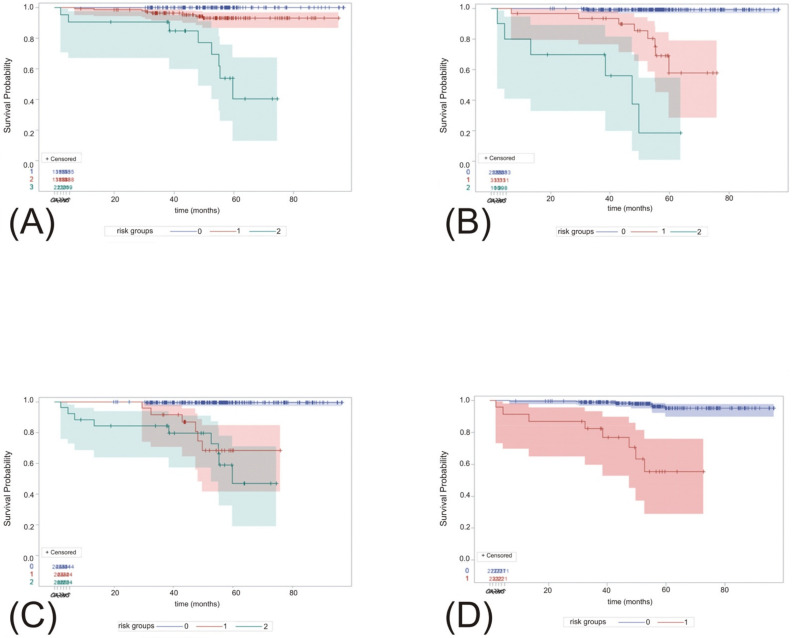
Kaplan–Meier curves representing cancer-specific survival according to the risk stratification as determined by our classification (**A**), and Leibovich (**B**), VENUSS (**C**) and GRANT (**D**) models. *p*-Values < 0.0001 were reached in all the respective models between the risk groups. Respective risk groups (0–2) were presented in different colors (**A**–**D**).

Cancer-specific survival was significantly different among individuals stratified according to the respective model risk groups, as presented in Figure 1.

### 3.7. Overall Survival According to Risk Stratification in Local and External Models

**Figure 2:**
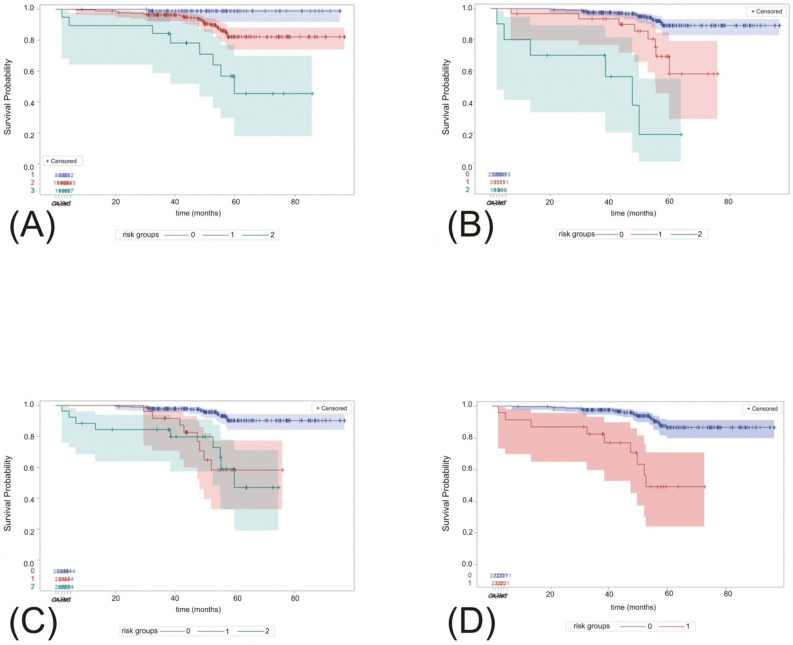
Kaplan–Meier curves representing overall survival according to the risk stratification as determined by our classification (**A**), and Leibovich (**B**), VENUSS (**C**) and GRANT (**D**) models. *p*-Values < 0.0001 were reached in all the respective models between the risk groups. Respective risk groups (0–2) were presented in different colors (**A**–**D**).

The respective subgroups of all models, including our classification, were proved to be significantly associated with OS (Figure 2).

### 3.8. External Validation of the Established Risk Models (Leibovich, VENUSS and GRANT) and Comparison with Our Model

**Figure 3:**
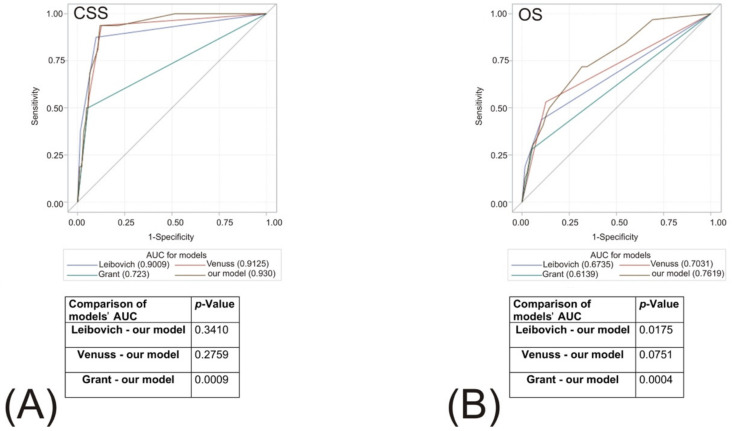
External validation of the previously established risk models: Leibovich, VENUSS and GRANT and comparison with our model. ROC curves for all the models were shown in different colors, representing prognostic values of the respective models for overall survival (**A**), and cancer-specific survival (**B**) (c-indexes of the models were provided in brackets). Additional collation with our model was presented with the respective *p*-Values.

Using receiver operating curves (ROCs), we performed an analysis of the performance of the already used clinical models for non-metastatic disease (Figure 3A,B). Our model demonstrated higher accuracy in terms of OS prediction compared to Leibovich and GRANT and outperformed GRANT in CSS prediction, while non-inferiority to VENUSS with respect to both endpoints was revealed. External validation of the above-mentioned models indicated their high accuracy in terms of CSS prediction and moderate accuracy in terms of OS prediction.

## 4. Discussion

In the current paper, we revisited the idea of using prognostic models in ccRCC with the incorporation of easily obtainable clinical factors that increase the prognostic properties to be used in a localized setting. Thus, the predictive value of tumor stage, size and grade was exploited, with the inclusion of CCI and novel hematological biomarkers, i.e., either SIRI or SII, depending on the end-point assessed. These features were incorporated into four-feature models, predicting either OS or CSS in localized ccRCC with increased accuracy when compared with three well-recognized models used in non-metastatic disease.

The constant search for an optimal tool to determine the scheme of follow-up after radical treatment, taking into consideration the risk of recurrence and survival, is just one perspective. The schedule includes risk, timing and the site of recurrence, which, in turn, imposes close monitoring in high-risk disease [9]. In the light of growing evidence on the efficacy of adjuvant treatment, the personalization of therapy using models for localized disease is the other side of the coin [10]. Clinical tools to assess patients may be even more sought after in order to determine possible high-risk candidates for future adjuvant treatment, e.g., in the paper by Choueiri et al. summarizing the results of the KEYNOTE-564 trial, adjuvant immunotherapy resulted in a remarkable increase in disease-free survival in high-risk patients [17].

There is no consensus established regarding the optimal risk stratification policy in localized RCC, although a variety of prognostic models are available [9,10,11,14,18,19]. While awaiting results of clinical trials on the role of perioperative systemic therapy, the appropriate selection of candidates may be vital. Taking into consideration the side effects and costs of this approach, validation and application of the risk-based hierarchy will be necessary to optimize and simplify inclusion criteria [18].

The question that arises is as follows: what is the true value of additional features that are included in the already established prognostic models in the light of ‘overfitting’ phenomenon during model creation? Only after providing the answer can one justify their everyday clinical application [20]. Furthermore, none of the existing models is routinely recommended based on its approved accuracy [14,19]. As a consequence, there is a need for balance between predictive accuracy and simplicity in practice: incorporating additional features may not result in better prognostic value, while it may make the tool too complicated.

Although it is obviously included in all the models, TNM staging proved to have limited accuracy if selected as a single prognostic factor [2]. Additional information has been routinely gathered from pathological examination, i.e., grading or presence of tumor necrosis or sarcomatoid features [2]. Here, we introduced a novel prognostic model developed in the contemporary ccRCC cohort with a stress on both overall and cancer-specific survival using easily approachable features. Firstly, we established significant predictors for CSS in multivariate analysis to be used in a future model. Apart from tumor stage, grade and size, we observed statistical significance for a single hematological marker, i.e., SII. On the other hand, in the multivariate analysis of OS we determined that next to tumor grade and size CCI and SIRI were significant predictors of survival. Berger et al. pinpointed that coexisting chronic diseases remain significant prognostic factors for overall survival after nephrectomy [21]. Collecting the relevant information and translating it into a validated score may increase the efficacy of perioperative evaluation of the candidates for surgery, as discussed by Charlson et al. [16]. It seems reasonable then to opt for the incorporation of the Charlson Comorbidity Index into the prognostic tools in the hope of achieving more personalized approaches [22]. We determined that CCI (>4 vs. ≤4) was a significant predictor in both univariate and multivariate analyses of OS but not CSS. Although a detailed description of patient comorbidities is a routine preoperative work-up to establish both perioperative risk and to define the benefits of invasive treatment, it is rarely taken into consideration, when survival after the nephrectomy is analyzed [3]. Santos Arrontes et al. found that a significant predictor of OS was not only stage but also CCI (discrimination ≤ 2 and >2) [23], while Ather et al. observed a feature of >5 CCI to be an independent predictor of OS in cases treated with either radical or partial nephrectomy for RCC [3]. On the other hand, one should be familiar with another finding i.e., Gettman et al. failed to confirm a similar association between CCI and CSS in a cohort of selected patients with venous tumor thrombus and emphasized the TNM of the primary lesion as of greatest importance [24]. It seems, however, that OS in RCC is not only tumor-dependent but also patient-dependent, as relevant factors including individuals’ comorbidities, gender and age should be acknowledged. Here, we found that age but not gender was significantly associated with survival in univariate analyses of both endpoints. However, we failed to incorporate it into the further multivariate analyses. It is consistent with the nationwide cohort study (n = 7894 participants) that pinpointed the relationship between survival of patients with RCC and comorbidities (cases with CCIs of 1–2 and ≥3 were found to have increased mortality rates when compared with patients with no defined comorbidity), regardless of age [25].

Recently, we found that the highest c-indexes were found when including SIRI or, alternatively, SII and NLR in the prognosis of localized RCC [13]. However, in the present population of ccRCC patients with a longer follow-up, only SIRI and SII reached statistical significance. In a recent paper by Mao et al., elevated SIRI was a better predictor of worse OS and CSS than LMR and hemoglobin [12]. Then, based on their own results, Lv et al. claimed that enforcing prognostic models with preoperative SIRI results in increased accuracy for RCC with tumor thrombus [26]. Hu et al. observed that high SII was found in cases with worse OS and CSS in a non-metastatic RCC cohort post-nephrectomy (n = 646) [27]. Ozbek et al. reported that elevated SII was found in patients with poor OS, but no association was revealed for disease-specific survival, despite the use of different thresholds [28]. Finally, in a meta-analysis that included 3180 RCC cases, Jin et al. reported that elevated SII was a strong indicator of poor OS (and aggressive disease) but not progression-free survival/disease-free survival or CSS [29]. These findings may shed some light on the associations of SIRI with OS and SII with CSS.

Interestingly, we failed to confirm associations with uptake of common drugs, including statins. This is consistent with recent papers, including a nationwide case–control study from Denmark (n = 4606 participants) [30]. Pottegard et al. did not confirm the hypothesis of a chemopreventive effect of long-term statin use on the development of RCC. On the other hand, Berquist reported that statin use resulted in improved CSS and OS [31].

Here, we validated three well-recognized models (VENUSS, GRANT and Leibovich) in our cohort and subsequently proceeded with the preparation of our own predictive tool based on clinicopathological features, including CCI and single inflammatory markers. Clearly, there is no single best model for all the populations of RCC that could be used in the assessment of all outcomes, i.e., OS, CSS and RFS [5]. In our model, we focused on localized ccRCC cases in the hope that we might determine other features that would have better discriminative properties when compared with TNM staging only. In the light of the multivariate analyses presented above, we described four-feature models for OS and CSS, respectively. Using ROC analyses we found that our model outperformed Leibovich and GRANT with respect to OS prognosis and GRANT with respect to CSS. On the other hand, we confirmed the non-inferiority of our model when compared with VENUSS. Therefore, external validation of the model would allow us to incorporate it into clinical applications, e.g., in enrollment for clinical trials purposes. Importantly, we discriminated between ≤T2 and ≥T3, high- vs. low-grade tumors and tumor sizes <7 or ≥7 cm, so Tumor characteristics were considered not only through T stage, while no additional pathological assessment was necessary (e.g., sarcomatoid features). The strength of the model may also lie with the incorporation of hematological biomarkers of established accuracy in the prognosis of RCC. Our model is not based on subjective clinical variables such as performance status but on intuitive calculations of CCI. Its validation in terms of predictive accuracy will enable its application in the adjuvant setting for high-risk patients treated with radical surgery to estimate the inclusion criteria for individuals that would gain benefit from systemic treatment. Finally, novel models and risk calculators can find their place in the field of transplantology, both during recipient qualification and the acceptance of organs with small renal lesions frequently found during donor assessment [32]. Our model may be of special interest to transplant clinicians due to the incorporation of blood count derivatives.

A principal limitation of our model establishment is the retrospective nature of the data from a single tertiary center. However, we focused on records that were complete for all patients and used a single pathological laboratory, a single laboratory for blood-count analyses and a single tool for CCI calculations. Additionally, the TNM classification that we used was based on the 2017 consensus [15], yet we included pathological grading according to Fuhrman and/or WHO/ISUP when adequate. Moreover, although the sample size was relatively small, we managed to obtain a satisfactory duration of follow-up with a standardized scheme. Our model, similar to other predictive models, is characterized by a significant deterioration in its performance over time. Furthermore, the outcome data were mainly based on intermediate- and low-risk patients. Finally, without external validation, we cannot exclude the possibility of model overfitting because of variable and threshold selections. Therefore, the prospective evaluation of our model in a larger population would enable its clinical application.

## 5. Conclusions

In conclusion, four different features were included in a model predicting the CSS (grade, size, stage and SII) and OS (grade, size, CCI and SIRI) of patients with localized non-metastatic ccRCC, characterized by adequate or even superior accuracy when compared with the VENUSS [9], Grant [10] and Leibovich [11] prognostic tools. The described scoring system for the risk of cancer-specific death and overall mortality can be used to stratify patients into respective risk groups for follow-up establishment or enrollment into clinical trials after prospective validation in a large population.

## Figures and Tables

**Table 1:** General characteristics of the studied cohort of patients with clear cell renal cell carcinoma (ccRCC).

Characteristics		No./Median	%/IQR
Gender	Female	109	37.07
	Male	185	62.93
Age		63	55–70
BMI	kg/m^2^	27.7	24.4–30.7
Stage	T1	246	83.67
	T2	20	6.80
	T3	26	8.84
	T4	2	0.68
Grade	1–2	254	86.39
	3–4	40	13.61
Charlson Comorbidity Index		4	3–5
CCI	≤4	169	57.48
	>4	125	42.52
Tumor diamater	<7 cm	247	84.01
	≥7 cm	47	15.99
SIRI	>2.15	170	57.82
	≤2.15	124	42.18
SII	>660	189	64.29
	≤660	105	35.71
Surgical treatment	RN	101	34.35
	NSS	193	65.65
Surgical modality	Lumbotomy	155	53
	Laparotomy	62	21
	Laparoscopy	77	26
Diabetes	Yes	39	13.36
	No	253	86.64
Hypertension	Yes	180	61.43
	No	113	38.57
Heart disease	Yes	38	13.01
	No	254	86.99
Autoimmune diseases	Yes	14	4.79
	No	278	95.21
Past MI	Yes	17	5.82
	No	275	94.18
Statins	Yes	56	19
	No	176	59.86
	Unknown	62	21.14
Beta-blockers	Yes	80	27.2
	No	152	51.7
	Unknown	62	21.1
GRANT risk group	Favourable	271	92.18
	Unfavourable	23	7.82
Leibovich risk group	Low	253	86.05
	Intermediate	31	10.54
	High	10	3.40
VENUSS risk group	Low	244	82.99
	Intermediate	24	8.16
	High	26	8.84
Outcomes			
Recurrence	No	253	86.05
	Yes	41	13.95
Death	No	262	89.12
	Yes	32	10.88
Cancer Death	No	278	94.56
	Yes	16	5.44

**Table 2:** Univariate analyses of factors predictive for cancer-specific survival in patients with clear cell renal cell carcinoma (ccRCC).

Factors Predicting Cancer-Specific Survival—Univariate Analyses					
Variables	Reference	OR	LL 95% CI	UL 95% CI	*p*-Value
Age	>60 vs. ≤60	3.057	0.852	10.969	0.0866
Gender	Male vs. female	0.437	0.158	1.209	0.1108
Charlson Comorbidity	>4 vs. ≤4	2.362	0.835	6.683	0.1052
SII	High vs. low	5.968	1.873	19.012	0.0025
SIRI	High vs. low	4.446	1.399	14.137	0.0115
Tumor grade	High- vs. low-grade	10.244	3.564	29.450	<0.0001
Stage T3–T4	T1–T2	17.526	5.880	52.236	<0.0001
Tumor size	≥7 cm vs. <7 cm	20.829	6.363	68.176	<0.0001
Surgery type	NSS vs. NR	0.107	0.030	0.385	0.0006
Hypertension	Yes vs. no	1.406	0.475	4.157	0.5382
Diabetes	Yes vs. no	0.418	0.054	3.253	0.4045
Statins	Yes vs. no	0.273	0.034	2.161	0.2186
Beta-adrenolytics	Yes vs. no	0.619	0.163	2.354	0.4816

**Table 3:** Multivariate analyses of factors predictive for cancer-specific survival in patients with clear cell renal cell carcinoma (ccRCC).

Factors Predicting Cancer-Specific Survival—Multivariate Analysis					
Variables	Reference	OR	LL 95% CI	UL 95% CI	*p*-Value
Stage T3–T4	T1–T2	5.101	1.414	18.396	0.0128
Tumor grade	High- vs. low-grade	3.948	1.099	14.188	0.0354
Tumor size	≥7 cm vs. <7 cm	6.420	1.693	24.351	0.0063
SII	High * vs. low	4.547	1.196	17.280	0.0262

* High defined as SII > 660.

**Table 4:** Univariate analyses of factors predictive for overall survival in patients with clear cell renal cell carcinoma (ccRCC).

Factors Predicting Overall Survival—Univariate Analyses					
Variables	Reference	OR	LL 95% CI	UL 95% CI	*p*-Value
Age	>60 vs. ≤60	2.625	1.096	6.286	0.03
Gender	Male vs. female	1.141	0.528	2.467	0.73
Charlson Comorbidity	>4 vs. ≤4	2.151	1.019	4.542	0.045
SII	High vs. low	2.241	1.069	4.697	0.033
SIRI	High vs. low	2.947	1.364	6.368	0.006
Tumor grade	High- vs. low-grade	4.209	1.844	9.606	0.0006
Stage T3–T4	T1–T2	5.005	2.033	12.324	0.0005
Tumor size	≥7 cm vs. <7 cm	4.588	2.078	10.132	0.0002
Surgery type	NSS vs. NR	0.416	0.198	0.874	0.02
Hypertension	Yes vs. no	0.908	0.430	1.919	0.80
Diabetes	Yes vs. no	0.402	0.092	1.753	0.23
Statins	Yes vs. no	0.687	0.247	1.906	0.47
Beta-adrenolytics	Yes vs. no	0.778	0.324	1.865	0.57

**Table 5:** Multivariate analyses of factors predictive for overall survival in patients with clear cell renal cell carcinoma (ccRCC).

Factors Predicting Overall Survival—Multivariate Analysis					
Variables	Reference	OR	LL 95% CI	UL 95% CI	*p*-Value
Tumor grade	High- vs. low-grade	2.964	1.135	7.740	0.0265
Charlson Comorbidity	>4 vs. ≤4	2.473	1.095	5.583	0.0293
Tumor size	≥7 cm vs. <7 cm	3.179	1.245	8.116	0.0156
SIRI	High * vs. low	2.453	1.073	5.609	0.0334

* High defined as SIRI > 2.15.

**Table 6:** Multivariate analyses of factors predictive for recurrence-free survival in patients with clear cell renal cell carcinoma (ccRCC).

Factors Predicting Recurrence-Specific Survival—Multivariate Analysis					
Variables	Reference	OR	LL 95% CI	UL 95% CI	*p*-Value
Tumor grade	High- vs. low-grade	3.373	1.473	7.719	0.0040
Tumor size	≥7 cm vs. <7 cm	3.605	1.634	7.954	0.0015

**Table 8:** Risk groups for cancer-specific death and all-cause mortality after surgical treatment of clear cell renal cell carcinoma.

	CSD Scoring		OM Scoring	
Risk Group	No. Pts.	%	No. Pts.	%
Low	151	51.36	82	27.89
Intermediate	122	41.50	193	65.65
High	21	7.14	19	6.46

## Data Availability

The data presented in this study are available on request from the corresponding authors.
